# Exploring the Prevalence and Factors Influencing Clinical Trial Awareness in US Adults with Self-Reported Depression and Anxiety

**DOI:** 10.7759/cureus.40780

**Published:** 2023-06-22

**Authors:** Ibilola A Sanusi, Abimbola E Arisoyin, Shaw Aruoture, Ibrahim L Folorunsho, Obiamaka P Okereke, Damilola A Adeyemo, Mujeeb A Salawu, Okelue E Okobi, Akash Gupta, Henrietta S Akunne, Radhey Patel, Omotola Emmanuel, Nneka C Ezeudemba

**Affiliations:** 1 Internal Medicine, University of Texas at Houston, Houston, USA; 2 Internal Medicine, College of Medicine, University of Lagos, Lagos, NGA; 3 Psychiatry, Behavioral Hospital Of Bellaire, Houston, USA; 4 Emergency Department, Bader Al Janoub General Hospital, Najran, SAU; 5 Family Medicine, Ebonyi Sate University, Ebonyi, NGA; 6 Research, Texas A&M University, Corpus Christi, USA; 7 Medicine and Surgery, University of Ilorin College of Health Sciences, Ilorin, NGA; 8 Internal Medicine and Psychiatry, Houston Health Department, Houston, USA; 9 Family Medicine, Medficient Health Systems, Laurel, USA; 10 Family Medicine, Lakeside Medical Center, Belle Glade, USA; 11 Internal Medicine, Spartan Health Sciences University, Vieux Fort, LCA; 12 Psychiatry, Delta State University, Abraka, NGA; 13 Psychiatry and Behavioral Sciences, Avalon University School of Medicine, Willemstad, CUW; 14 Health Information Management, Emory Healthcare, Georgia, USA; 15 Infectious Disease, Medical University of Silesia, Katowice, POL

**Keywords:** mental health, united states, clinical trial engagement, clinical trial awareness, anxiety, depression

## Abstract

Objective: Lack of clinical trial awareness is a crucial barrier to clinical trial enrollment. The objective of this study was to examine the prevalence and factors associated with clinical trial awareness among US adults with self-reported depression and anxiety.

Methods: Data were collected from 896 adults who self-reported depression and anxiety from the 2020 Health Information National Trends Survey. Multinomial logistic regression was utilized to assess predictors of clinical trial awareness, particularly socio-demographic, health-related, and technological variables. Odds ratios (OR) for the associations were reported.

Results: About 60.4% of adults with self-reported depression or anxiety reported being aware of clinical trials. In the multivariable regression, education level, health-related social media use, and having access to a regular provider were all significantly associated with greater odds of clinical trial awareness among individuals with depression and/or anxiety. Specifically, individuals with at least some college education (OR 2.07, 95% confidence interval (CI); 1.28-3.34; p ​= ​0.004) were more likely to report awareness of clinical trials than those with less than a college education. Similarly, compared to those without access to health providers, individuals with depression and/or anxiety who had a regular provider had greater odds of clinical trial awareness (OR 2.23, 95% CI; 1.16-4.31; p ​= ​0.017). Additionally, those who reported two or more health-related uses of social media were significantly more likely to report clinical trial awareness than their counterparts who reported no health-related social media use (OR 3.17, 95% CI; 1.48-6.80; p ​= ​0.004).

Conclusion: Our study shows that about six in 10 adults with depression and anxiety in the United States were aware of clinical trials. However, some sub-groups of patients, particularly those without access to a regular health provider, those with a lower education level, and those with limited use of social media for health purposes, remain inadequately informed and may lack awareness of available clinical trials. These findings are crucial and identify subgroups of people with mental disorders that may benefit from targeted interventions to improve clinical trial awareness.

## Introduction

Mental disorders, including depression and anxiety, affect millions of people and account for a significant portion of the global burden of disease morbidity and mortality. Evidence from the National Institute of Mental Health (NIMH) shows that almost 10% of US adults (21 million people) reported at least one depressive episode in 2020 alone [1]. Similarly, the Centers for Disease Control and Prevention (CDC) reported that in 2019, 11.2% reported regular feelings of worry, nervousness, or anxiety [2]. Worryingly, there is sufficient evidence indicating that the prevalence of these mental disorders may have increased significantly due to the coronavirus pandemic [3-6]. In one report, researchers found that between August 2020 and February 2021, there was an increase in the proportion of adults reporting recent symptoms of anxiety or depression from 36.4% to 41.5% [6]. Evidently, these substantially high rates of depression and anxiety pose a significant challenge to the health system and underscore the need to develop effective mental health treatments.

Clinical trials remain the foundation for the advancement of innovations in science and medicine. Clinical trials offer the opportunity to evaluate the efficacy and safety of innovative interventions, leading to the development of novel treatments and improvements in mental health care. The relevance of clinical trials to advancing mental health treatments has been well documented. For example, the Sequenced Treatment Alternatives to Relieve Depression (STAR*D) trial was a landmark trial that offered useful guidance on the real-world effectiveness of anti-depressant medications. Trials such as STAR*D highlight the potential impact of clinical trials on mental health treatments and help quantify the potential impact of a study [7]. Other large-scale trials for different psychiatric populations have also been conducted, with exciting and promising findings that have ushered in new frontiers for treatment [8,9].

Despite the benefits of clinical trials in improving mental health outcomes, rates of patient enrollment in clinical trials continue to be low [10,11], with resultant implications for the pace of clinical innovation as well as the generalizability of trial findings. Lack of clinical trial awareness has been widely reported as a crucial barrier to clinical trial participation and may be particularly relevant in mentally ill populations, who are underrepresented and often excluded from clinical trials [12].

Although numerous studies have examined clinical trial awareness in various clinical populations, there is a noticeable gap in the literature concerning awareness, specifically among individuals with mental disorders. Previous research has primarily focused on clinical trial awareness among cancer patients and minority populations [13-15]. While these studies have provided valuable insights into factors influencing clinical trial awareness, it is essential to explore this area within the context of mental health disorders, given the unique challenges and considerations associated with psychiatric conditions. Currently, there is limited information regarding the awareness of clinical trials among individuals with mental disorders. Understanding the prevalence and factors associated with clinical trial awareness is critical for several reasons. Firstly, awareness is a crucial prerequisite for individuals to actively engage in clinical trial participation. Secondly, disparities in clinical trial awareness can lead to unequal access and potentially exacerbate existing health inequities. Identifying factors associated with awareness will enable targeted interventions to ensure equitable dissemination of information across diverse populations. Lastly, by uncovering the level of awareness and factors associated with it, this study can provide insights into potential strategies for improving communication and education about clinical trials among individuals with mental disorders.

Accordingly, using the Health Information National Trends Survey, this study sought to examine the prevalence and factors associated with clinical trial awareness among individuals with mental disorders in the United States, specifically those with depression and/or anxiety. A nuanced understanding of the predictors of clinical trial awareness can help inform campaigns to improve clinical trial participation in both mental health contexts [16].

## Materials and methods

Study design

We examined participant data from the Health Information National Trends Survey (HINTS) 5 Cycle 4 (H5C4), conducted from February 24 through June 15, 2020, by the National Cancer Institute (NCI) via mailed questionnaires. HINTS 5 is a nationally representative survey in English administered every three years to non-institutionalized civilian adults (aged 18 years) living in the United States to assess knowledge of, attitudes toward, and use of health information. HINTS data are publicly available, de-identified, and approved by the Westat Institutional Review Board. Therefore, our study is considered exempt from our local institutional review board's assessment or review by the US National Institute of Health Office of Human Subjects Research Protections. Information on the HINTS methodology, sampling, and data collection details has been published elsewhere [16]. We followed the Strengthening the Reporting of Observational Studies in Epidemiology (STROBE) guidelines [17].

Briefly, the H5C4 employed a two-stage, stratified random sampling technique. The first stage involves the selection of non-vacant residential addresses obtained from the Marketing Systems Group (MSG). In the second stage, an adult from each household was selected for participation in the survey using the Next Birthday method. The database of residential addresses was then grouped into two categories: high-minority strata (areas with ≥34% Hispanics or African Americans) and low-minority strata (areas with <34% Hispanics or African Americans). This stratification was done to increase the precision of estimates for minority subpopulations. The survey respondents were then weighted to reflect selection probabilities and to provide a nationally representative sample in terms of age, gender, educational attainment, marital status, race, ethnicity, and census region. In addition to the full-sample weight, a set of 50 replicate weights was provided for each adult. These replicate weights are used to calculate the standard error of estimates obtained from the HINTS data using the delete one jackknife (JK1) replication method [18].

Measures

Clinical trial awareness was the dependent variable. Participants were asked, "How would you describe your level of knowledge about clinical trials?". Response options were "I don’t know anything about clinical trials," "I know little about clinical trials," or "I know a lot about clinical trials." For the purpose of this analysis, we dichotomized these responses into having clinical trial knowledge ("I know a little" or "I know a lot about clinical trials") and not having any knowledge of clinical trials ("I don’t know anything about clinical trials").

Sample population

Self-Reported Diagnosis of Depression or Anxiety

Similar to past research [19-21], individuals with depression and anxiety were determined using the answer of the participant to the question, "Has a doctor or other health professional ever told you that you had depression or anxiety disorder (yes/no)?"

Participant Characteristics and Covariates

Covariates included in this study were informed by prior literature on factors associated with clinical trial awareness [22]. The specific sociodemographic and health-related variables included in the present study were age (18 to 34 years, 35 to 49 years, 50 to 64 years, and 65 years or older), sex, race or ethnicity (White, Black or African American, Hispanic, and others), educational level (less than college, some college or college graduate or postgraduate), household income (less than $20k, $20k to less than $35k, $35k to less than $50k, $50k to less than $75k, and $75k or above), having a regular provider, previous cancer diagnosis, health-related social media use, and rural-urban residence. Health-related social media usage was derived in response to the following survey questions: "In the last 12 months, (i) Have you shared health information on social networking sites, such as Facebook or Twitter? (ii) Have you participated in an online forum or support group for people with similar health or medical issues? (iii) Have you watched a health-related video on YouTube?". The response options for all the question items were "yes" and "no," and thus they were dichotomized. We created a composite variable to categorize health-related social media use. Participants who endorsed one and more than one health-related social media use were categorized as social media users (and coded as one and two, respectively), and those reporting no health-related social media use were characterized as non-users (coded as 0).

Statistical analysis

All statistical analyses were completed using the "svy" command in the Stata 17.0 statistical software (StataCorp LP, College Station, Texas, USA). Final person weights and jack-knife replicate weights provided within the H5C4 dataset were used to derive national-level estimates and associated standard errors, respectively [16] Descriptive statistics using chi-squared tests were conducted for the entire study sample. Frequencies and weighted percentages were estimated for the overall adult population by age, sex, annual household income, race or ethnicity, level of education, having a regular provider, previous cancer diagnosis, health-related social media use, and rural or urban residence. Multivariable logistic regression analyses were conducted to examine the factors associated with knowledge of clinical trials among the study population. The regression modes controlled for sociodemographic and health-related variables, and statistical significance was set at p<0.05.

## Results

Sample characteristics

The final study sample included 896 respondents representing 60,796,771 individuals aged 18 years or older who self-reported depression and anxiety and provided responses to survey items enquiring about awareness of clinical trials. About 58.6% were aged between 18 and 49 years, 62% were female, 69.2% were non-Hispanic White, 64.3% had some college or higher education, 9.0% reported having ever heard of the clinicaltrials.gov website, and 69.3% reported having access to a regular health provider. Overall, approximately 60.4% of the individuals with depression or anxiety reported being aware of clinical trials.

In bivariate analysis (Table 1), among the study population, those who reported awareness of clinical trials were more likely to be people with higher education (67.9% had attended at least some college or higher level of education vs. people with less than a college education (47.1%)), people who had heard about the clinicaltrial.gov website (85.9%) vs. people who had not (59.3%), people with access to a regular health provider (64.9%) vs. people without a regular provider (49.6%), and people with two or more health-related social media usage (76.6%) vs. people with only one health-related social media use (61.9%) or people with no health-related social media usage (51.7%). Awareness of clinical trials did not differ by income, race, geographic residence, age, or gender. Regarding race, 45.5% of Hispanics, 63.7% of Whites, and 53.0% of Blacks reported awareness of clinical trials.

**Table 1:** Sociodemographic characteristics by awareness of clinical trials among the sample (-) = Intentional empty cell

Demographic variables	Total (N = 896), %	Awareness of clinical trials: No (N = 291), %	Awareness of clinical trials: Yes (N = 605), %	p-value
Gender	-	-	-	0.601
Female	62.0	40.2	59.8	
Male	38.0	37.0	63.0	
Age group (years)				0.807
18-34	27.7	41.0	59.0	
35-49	30.9	41.4	58.6	
50-64	27.4	35.6	64.4	
65+	14.0	42.7	57.3	
Education	-	-	-	<0.001
Less than college	35.7	52.9	47.1	
At least some college	64.3	32.1	67.9	
Household Income	-	-	-	0.400
$20,000	27.6	45.8	54.2	
$20,000 - $34,999	10.5	44.6	55.4	
$35,000 - $49,999	11.2	43.2	56.8	
$50,000 - $74,999	15.0	33.3	66.7	
$75,000 or more	35.7	34.6	65.4	
Race	-	-	-	0.187
White	69.2	36.3	63.7	
Black/African American	10.4	47.0	53.0	
Hispanic	13.3	54.5	45.5	
Others	7.1	35.2	64.8	
Ever had cancer status				0.230
No	90.6	40.7	59.3	
Yes	9.4	32.7	67.3	
Residence	-	-	-	0.409
Urban	87.5	38.6	61.4	
Rural	12.5	46.2	53.8	
Ever heard of the website clinicaltrials.gov?	-	-	-	0.005
No	91.0	40.7	59.3	
Yes	9.0	14.1	85.9	
Regular Provider				0.035
No	30.7	50.4	49.6	
Yes	69.3	35.1	64.9	
Health-related social media use	-	-	-	0.002
None	45.1	48.3	51.7	
One form	33.6	38.1	61.9	
Two or more forms	21.3	23.4	76.6	

In the multivariable regression (Table 2), education level, health-related social media use, and having access to a regular provider were all significantly associated with greater odds of clinical trial awareness among individuals with depression and/or anxiety. Specifically, individuals with at least some college education (odds ratio (OR) 2.07, 95% confidence interval (CI); 1.28-3.34; p ​= ​0.004) were more likely to report awareness of clinical trials than those with less than a college education. Similarly, compared to those without access to health providers, individuals with depression and/or anxiety who had a regular provider had greater odds of clinical trial awareness (OR 2.23, 95% CI; 1.16-4.31; p ​= ​0.017). Additionally, those who reported two or more health-related uses of social media were significantly more likely to report clinical trial awareness than their counterparts who reported no health-related social media use (OR 3.17, 95% CI; 1.48-6.80; p ​= ​0.004).

**Table 2:** Multivariable logistic regression for the factors associated with clinical trial awareness among individuals with depression and/or anxiety (-); Intentional empty cell

Demographic variables	Awareness of clinical trial: Adjusted odds ratio, 95% confidence interval	p-value
Gender	-	-
Female	1.00	
Male	0.91 (0.55, 1.49)	0.697
Age Group (years)	-	-
18-34	1.00	-
35-49	1.03 (0.42, 2.48)	0.953
50-64	0.98 (0.41, 2.34)	0.955
65+	1.07 (0.36, 3.18)	0.901
Education		
Less than college	1.00	-
At least college	2.07 (1.28, 3.34)	0.004
Household Income	-	-
$20,000	1.00	-
$20,000 - $34,999	1.08 (0.37, 3.14)	0.885
$35,000 - $49,999	1.08 (0.41, 2.89)	0.871
$50,000 - $74,999	1.45 (0.57, 3.72)	0.429
$75,000 or more	1.18 (0.56, 2.46)	0.659
Race	-	-
White (reference)	1.00	-
Black/African American	0.59 (0.26, 1.36)	0.211
Hispanic	0.72 (0.27, 1.95)	0.512
Others	0.73 (0.25, 2.19)	0.572
Ever had cancer status	-	-
No (reference)	1.00	-
Yes	1.65 (0.84, 3.25)	0.140
Residence	-	-
Urban (reference)	1.00	-
Rural	0.52 (0.20, 1.30)	0.156
Ever heard of the website clinicaltrials.gov?	-	-
No (reference)	1.00	-
Yes	3.00 (0.77, 11.8)	0.111
Regular provider	-	-
No (reference)	1.00	-
Yes	2.23 (1.16, 4.31)	0.017
Health-related social media use	-	-
None (reference)	1.00	-
Only 1 form	1.30 (0.66, 2.54)	0.437
Two or more forms	3.17 (1.48, 6.80)	0.004

## Discussion

Generally, this study sought to expand the literature on clinical trial awareness in mental health contexts. To do so, we drew on data from the 2020 Health Information National Trends Survey to ascertain the levels of clinical trial awareness and explore associations with sociodemographic and health-related characteristics among a nationally representative sample of people with self-reported depression and anxiety. Two major themes emerged from our analysis: i) about six in 10 (60.4%) of adults with self-reported depression and/or anxiety were aware of clinical trials, and ii) awareness differed according to sociodemographic, health-related, and technological factors.

To the best of our knowledge, this is one of the first studies investigating the levels of clinical trial awareness among those with mental disorders. The finding that only 60% of adults with depression and/or anxiety were aware of clinical trials is below the higher levels (72.9%) that have been reported in the general US population [22]. While no past studies on clinical trial awareness in psychiatric populations exist for comparison, the suboptimal levels of clinical trial awareness reported in our population of adults with depression and/or anxiety underscore the need to develop strategies and programs to improve awareness of clinical trials in this highly vulnerable group.

Regarding sociodemographic factors, one key finding from our analysis is that educational background was independently associated with clinical trial awareness. Our observation that the odds of clinical trial awareness increased with higher levels of education among those with mental disorders is consistent with findings from previous research in the general population [22,23]. Over the past decade, large-scale national educational efforts have been instituted to improve clinical trial awareness [24,25], and resultantly increase clinical trial participation rates in the general US population. While there is some evidence suggesting these educational efforts may have yielded some benefits [22], concerns remain that among people with mental disorders, those from low educational backgrounds continue to be left behind. Our findings demonstrate a need to focus efforts on mitigating this disparity and offer useful information that could guide the targeted implementation and effective integration of programs to promote clinical trial awareness among those with lower educational backgrounds.

In contrast to other past works [22,23], our findings suggest that among people with depression and/or anxiety, there were no racial or income differences in awareness of clinical trials. On the one hand, while these findings could indicate that the implementation of large-scale clinical trial awareness education programs [24,25] and a marked uptake in health-related internet use [26,27] in the United States may have resulted in narrowing the racial and income gaps in clinical trial awareness over time, it is imperative to mention that our study investigated the extent of clinical trial awareness during the COVID-19 pandemic. Thus, it is possible that heightened discussions around health care during the pandemic contributed to increasing awareness of clinical research studies, especially among historically underrepresented communities.

Importantly, we observed that having a regular health provider was associated with increased odds of being aware of clinical trials. This finding supports the notion that patient communication with a primary healthcare provider represents an important facet in raising awareness of clinical trials [28]. One plausible explanation for our result is that having a regular health provider facilitates a consistent and ongoing relationship with a healthcare professional, providing more opportunities for communication and information exchange. Thus, providers are likely to discuss treatment options, including clinical trials, with their patients, thereby increasing awareness. This line of thought is further supported by previous works showing that most patients view their health providers as their primary source of education and advice on clinical trials [29] and that physician input is critical to public engagement in clinical trials [30,31].

Emerging communicative technologies, such as social media, have the potential to educate and disseminate health care information to a wider audience. Social media may be useful in promoting clinical trial awareness among multiple clinical populations, including those with mental disorders. Our findings suggest that using social media for health-related information may increase awareness of clinical trials. In the final analysis, people with depression and/or anxiety were three times more likely to have heard of clinical trials if they used social media for health-related purposes than their counterparts who did not. Our observation aligns with previous research suggesting an increasingly important role for social media in clinical trials [32-34]. Past work also indicates that social media can serve as a platform for disseminating mental health information and promoting mental health awareness [35]. Also, a recent systematic review found that social media is increasingly employed in mental health research recruitment as it offers significant benefits in cost, speed, and efficiency [36]. Our analysis shows that, similarly, social media can be deployed to promote awareness of clinical trials in mentally ill populations and highlights social media as a potentially viable tool for targeted and specific communication designed to increase awareness of clinical trials in mental health contexts. Given that clinical trial awareness is a crucial barrier to clinical trial recruitment, the current study's findings are important and provide directions for future studies on the determinants of clinical trial awareness and literacy among mental health populations.

Strengths and limitations

The main strength of this current study is our use of a large, nationally representative cohort of US individuals with depression and/or anxiety. Our data offer insights and extend the literature on clinical trial awareness among adults with mental disorders (specifically depression and/or anxiety). Also, sampling weights enable us to generalize our findings to the entire US population.

Despite these strengths, our study has limitations. First, our study population consisted of individuals with a self-reported history of depression and/or anxiety. Due to the nature of the HINTS survey questions, we were not able to ascertain the severity of their symptoms or if they were receiving any mental health care. Thus, our findings cannot be extended to other mental health populations, such as those with serious mental illnesses like schizophrenia and bipolar disorder. Second, the survey item used to operationalize the clinical trial awareness variable was not specific, and it was not possible to determine if participants were responding to general clinical trials or if they were responding to their awareness of mental health trials. Third, the design of the survey questions limited our ability to examine other theoretical factors such as the provider-patient relationship and trust in doctors, as well as clinical trial awareness levels. Lastly, the data were cross-sectional and obtained by self-report, which might have introduced recall bias and precluded the ability to infer trends in awareness over time. While improving clinical trial awareness in mental health contexts is relevant, future studies should focus on exploring if improved awareness translates into actual clinical trial participation across a broader spectrum of mental health patients. It is also important to acknowledge that this research may not have accounted for various potential confounding factors that could have influenced the results. For instance, the influence of social support networks and their potential impact on the outcomes could have impacted the observed outcomes.

While there are unclear racial or income disparities, this gap may be narrowing. Technology, efforts to improve trial awareness by the government, and the heightened healthcare discussions during the pandemic and with the specific population may be reasons for our findings to be similar to earlier data. Our results provide early evidence that awareness may be improving, and more efforts should shift towards improving participation and outcomes, given that studies still show racial and ethnic disparities in participation among a unique population of people with mental illness [11, 22-27,37].

## Conclusions

Clinical trials remain pivotal in advancing treatments and improving outcomes for individuals with mental disorders. Our study shows that about six in 10 adults with depression and anxiety in the United States were aware of clinical trials. However, some sub-groups of patients, particularly those without access to a regular health provider, those with a lower education level, and those with limited use of social media for health purposes, remain inadequately informed and may lack awareness of available clinical trials. These findings are crucial and identify subgroups of people with mental disorders that may benefit from targeted interventions to improve clinical trial awareness.
